# Light-Mediated Control over TRPC3-Mediated NFAT Signaling

**DOI:** 10.3390/cells9030556

**Published:** 2020-02-27

**Authors:** Annarita Graziani, Bernadett Bacsa, Denis Krivic, Patrick Wiedner, Sanja Curcic, Rainer Schindl, Oleksandra Tiapko, Klaus Groschner

**Affiliations:** Gottfried-Schatz-Research-Center – Biophysics; Medical University of Graz, Neue Stiftingtalstrasse 6/D04, 8010 Graz, Austria; annarita.graziani@medunigraz.at (A.G.); bernadett.bacsa@medunigraz.at (B.B.); denis.krivic@medunigraz.at (D.K.); patrick.wiedner@medunigraz.at (P.W.); sanja.curcic@medunigraz.at (S.C.); rainer.schindl@meunigraz.at (R.S.)

**Keywords:** transient receptor potential channels, TRPC3 pharmacology, NFAT nuclear translocation, photochromic ligands, OptoBI-1

## Abstract

Canonical transient receptor potential (TRPC) channels were identified as key players in maladaptive remodeling, with nuclear factor of activated T-cells (NFAT) transcription factors serving as downstream targets of TRPC-triggered Ca^2+^ entry in these pathological processes. Strikingly, the reconstitution of TRPC-NFAT signaling by heterologous expression yielded controversial results. Specifically, nuclear translocation of NFAT1 was found barely responsive to recombinant TRPC3, presumably based on the requirement of certain spatiotemporal signaling features. Here, we report efficient control of NFAT1 nuclear translocation in human embryonic kidney 293 (HEK293) cells by light, using a new photochromic TRPC benzimidazole activator (OptoBI-1) and a TRPC3 mutant with modified activator sensitivity. NFAT1 nuclear translocation was measured along with an all-optical protocol to record local and global Ca^2+^ pattern generated during light-mediated activation/deactivation cycling of TRPC3. Our results unveil the ability of wild-type TRPC3 to produce constitutive NFAT nuclear translocation. Moreover, we demonstrate that TRPC3 mutant that lacks basal activity enables spatiotemporally precise control over NFAT1 activity by photopharmacology. Our results suggest tight linkage between TRPC3 activity and NFAT1 nuclear translocation based on global cellular Ca^2+^ signals.

## 1. Introduction

The pathological scenario of maladaptive structural remodeling is a pivotal process in the development of severe organ pathologies, specifically in the cardiovascular system [1,2,3]. Alterations in cellular Ca^2+^ homeostasis, prominently featuring enhanced Ca^2+^ entry based on G protein-coupled receptor signaling and activation of canonical transient receptor (TRPC) channels, have been recognized as a basis for rewiring transcriptional control in response to chronic stress [2,4,5]. TRPC3/6/7 pore complexes are able to sense various stress signals, and are primarily regulated by phospholipase C (PLC)-derived lipid mediators [6,7,8], and TRPC3 stands out for its distinct constitutive activity, which is reportedly governed by its glycosylation state [9]. TRPC3 as well as its closest relative TRPC6 have been identified to trigger Ca^2+^-dependent nuclear factor of activated T-cells (NFAT) nuclear translocation in native cardiovascular cells including myocytes [10,11,12] as well as fibroblasts [13]. Pharmacological abrogation of TRPC3/6 function was suggested as a potential therapeutic strategy for cardiovascular remodeling and heart failure prevention [14]. Although TRPC-mediated control of NFAT transcriptional activity has been repeatedly demonstrated in native cell systems, experiments reconstituting the coupling of TRPC signaling to NFAT nuclear translocation were inconclusive. While endogenous TRPC3 was shown to activate NFAT1 signaling in cardiac myocytes, recombinant TRPC3 was barely effective or even failed to trigger significant activation of NFAT1 when expressed in human embryonic kidney (HEK293) cells [15]. HEK293 cells have been extensively used to characterize Ca^2+^ channel functions and signaling mechanisms including cellular functions of TRPC channels. To this end, there is a lack of understanding of the principles underlying cellular decoding of TRPC-mediated Ca^2+^ signals and their translation into NFAT-governed transcriptional programs.

To shed light onto these issues, we took advantage of newly developed photochromic ligands that enable effective spatiotemporal control over cellular TRPC3 activity [16]. Moreover, the recent advancements in understanding structure-function relations in TRPC3 complexes revealed point mutations that cause distinct and subtle changes in channel function and can serve opto-chemogenetic approaches. Recently, a mutation in a TRPC3 pore domain (G652A) was found to generate super-sensitivity to small molecule activators harboring a benzimidazole moiety [17]. Moreover, the G652A mutation largely eliminates regulation of the channel by PLC-derived stimuli and abolishes its constitutive activity [18]. Thereby, the G652A mutation appears suitable for gaining full control over spatiotemporal TRPC3 activity pattern in an experimental setting of heterologous expression of channels combined with photopharmacology. With these tools at hand, we set out to explore whether light-mediated tuning of TRPC3 signaling enables control over NFAT1 activity in HEK293 cells. We report here for the first time proof of concept for precise manipulation of cellular NFAT activity by photopharmacological targeting of a TRPC channel. Moreover, our results suggest enhanced expression and basal activity of TRPC3 are linked to constitutive nuclear translocation of NFAT1, associated with distortion of cellular Ca^2+^ homeostasis. This chain of events is likely to contribute to initiation of tissue remodeling processes.

## 2. Methods

### 2.1. Reagents and Construct

All reagents used were of molecular biology grade, purchased from Merck Sigma-Aldrich (Vienna, Austria) unless specified otherwise. Mutation (G652A) in the hTRPC3 peYFP (WT-YFP; UniProt #Q13507, isoform 3) was introduced using a one-step site directed plasmid mutagenesis protocol [19]. Primers used for introduction of G652A peYFP (G652A-YFP) point mutation were as follows:

- Forward TTCTTTATGCAATATACAATGTAACTATGGTGGTCGTTTTACTCAA
- Reverse ATTGTATATTGCATAAAGAACGTATCCAATATTTTCTATGAATTTGTGATC.

CMV-R-GECO1.2 construct (#45494) was obtained from Addgene (Teddington, UK). Wild-type TRPC3 fusion with R-GECO 1.1 (WT–R-GECO) was generated by inserting the hTRPC3 gene using KpnI restriction sites into CMV-R-GECO1.2. Mutant TRPC3_G652A_ fusion with R-GECO 1.1 (G652A-R-GECO) was generated by using the one-step site directed plasmid mutagenesis protocol [19] and primers described above.

CFP-NFAT1 construct was kindly provided by Christoph Romanin (Johannes Kepler University, Austria). OptoBI-1 was synthetized at Bio-Techne (Bristol, GB).

### 2.2. Cell Culture and Genetic Manipulations

All experiments were performed in human embryonic kidney 293 (HEK293) cells and no special ethical considerations applied. Cells were cultured in Dulbecco’s modified Eagle’s medium (DMEM, Merck Sigma-Aldrich, Vienna, Austria) supplemented with 10% fetal bovine serum (FBS), streptomycin (100 μg/mL), penicillin (100 U/mL), l-glutamine (2 mmol/L), and HEPES (10 mmol/L). Cells were maintained in an incubator at 37 °C, 5% CO_2_. HEK293 cells grown on glass cover slips in 35 mm-dishes were transiently transfected at around 80% confluency with 2 μg plasmid DNA using PolyJet™ (SignaGen Laboratories, Rockville, USA) according to the manufacturer’s protocol. Experiments were performed 20–24 h after transfection.

### 2.3. Electrophysiology

**Figure 1:**
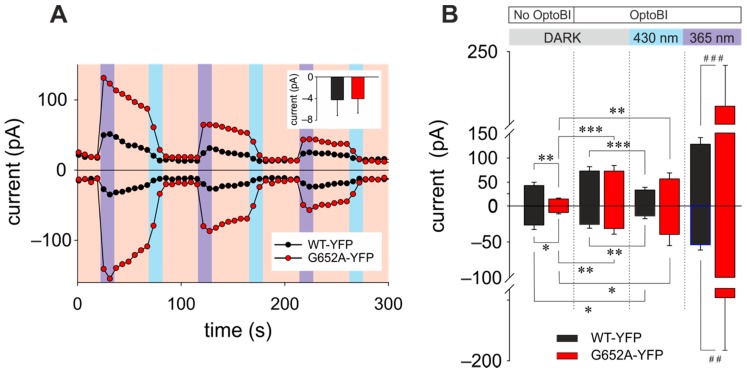
Manipulation of amplitude and temporal features of transient receptor potential canonical 3 (TRPC3) channel signaling patterns by photopharmacology and chemogenetics. (**A**) Representative time courses of the WT-YFP (white) and G652A-YFP (red) conductances recorded at −90 and 70 mV during repetitive photoconversion of OptoBI-1 (10 μM). Light illumination cycling is indicated as red (577 nm), UV (365 nm), and blue (430 nm). The insert shows mean ± SEM reversal potentials of currents through WT (black; *n* = 9) and G652A (red; *n* = 8) under bionic conditions ([Ca^2+^]_o_/[Cs^+^]_i_). (**B**) Basal (DARK), under blue and UV light application currents responses of WT-YFP and G652A-YFP in absence (No OptoBI) and presence of OptoBI-1 (10 μM). Currents obtained at −90 and 70 mV (mean ± SEM). Statistical significance was tested by two tailed t-test (normally distributed values, *) or Mann–Whitney tests (non-normally distributed values, #), * *p* < 0.05, ** *p* < 0.01, *** *p* < 0.001, if not indicated differences are not significant (*p* > 0.05).

Whole-cell electrophysiology was performed at room temperature. HEK293 cells were seeded on coverslips 24 h prior to the experiments. Coverslips were transferred to the patching bath on an inverted Axiovert 200 microscope (Zeiss, Oberkochen, Germany). WT-YFP and G652A-YFP transfected cells were identified by their yellow fluorescence when illuminated at 500 nm using Oligochrome light source (TILL Photonics FEI Company, Graefelfing Germany). Whole cell measurements were performed using an Axopatch 200B amplifier (Molecular Devices, Munich, Germany) connected with a Digidata-1440A Digitizer (Axon Instruments, Munich, Germany). Signals were low-pass filtered at 2 kHz and digitized with 8 kHz. Application of linear voltage-ramp protocol ranging from −130 to 80 mV (holding potential 0 mV) was controlled by Clampex 10.4 (Axon Instruments, Munich, Germany) software. Current densities at −90 and 70 mV were plotted against time and normalized by capacitance. Current–voltage relationships from −130 to 80 mV were normalized by capacitance. Illumination for photopharmacological measurements was switched every 10 s between UV (365 nm) and blue (430 nm) light. Patch pipettes were pulled from thin-wall filament glass capillaries (Harvard Apparatus, W3 30-0068, Holliston, USA), using a Sutter Instruments P1000 puller and BOX filaments FB245B to a resistance of 2–4 MΩ. Extracellular solutions contained (in mM): 140 NaCl, 10 HEPES, 10 Glucose, 2 MgCl_2_, 2 CaCl_2_, pH adjusted to 7.4 with NMDG. Pipette solution contained (in mM):120 cesium methanesulfonate, 20 CsCl, 15 HEPES, 5 MgCl_2_, 3 EGTA, titrated to pH 7.3 with CsOH. Osmolarity of all solutions was between 290 and 315 mOsm. Note that ionic conditions used in electrophysiological experiments varied from physiological condition in order to eliminate contaminating K^+^ currents and obtain a clear characterization of TRPC conductances. For Ca^2+^ permeability measurements, extracellular solution was modified as follows (mM): 132 NMDG, 10 HEPES, 10 glucose, 7 Ca D-gluconate, 3 CaCl_2_, 2 MgCl_2_, pH-adjusted to 7.4 with methanesulfonic acid. In pipette solution, 10 mM BAPTA were used instead of EGTA. Permeability ratios were calculated from reversal potentials (Figure 1A insert) using Goldman–Hodgkin–Katz equation modified for divalent ions and corrected for liquid junction potential.

### 2.4. [Ca^2+^]_i_ Imaging

Changes in intracellular Ca^2+^ ([Ca^2+^]_i_) were monitored using red-shifted genetically-encoded Ca^2+^ sensor (R-GECO1.2). Briefly, HEK293 cells overexpressing R-GECO, WT–R-GECO or G652A–R-GECO were washed twice with experimental buffer (EB composition in mM: 140 NaCl, 5 KCl, 1 MgCl_2_, 10 HEPES, 10 glucose and 1 CaCl_2_ (pH 7.4, adjusted with NaOH). The coverslip was then transferred to the imaging bath containing EB and 10 µM OptoBI-1 on an inverted microscope (Olympus IX71, Vienna, Austria) with 40 × 1.3 N.A. oil-immersion objective. Cells were excited to follow the R-GECO signal using 577/25 nm filters via TILL Oligochrome light source (TILL Photonics FEI Company, Graefelfing Germany) and fluorescent images were captured every second at 632 nm (using 632/60 nm emission filter, Chroma Technology, VT, USA) with an ORCA-03G digital CCD camera (Hamamatsu, Herrsching am Ammersee, Germany) using Live Acquisition 2.6 software (TILL Photonics FEI Company, Graefelfing Germany). The cis isomerization of OptoBI-1 compound was triggered by exposure to 10 s illumination period at 365 nm, and subsequent reversal of OptoBI-1 to trans conformation was achieved with 430 nm light exposure to 10 s period. The cycling between UV (365 nm; violet) and blue light (430 nm; blue) illuminations were employed one once or repeated three times. All experiments were performed at room temperature.

### 2.5. NFAT Nuclear Translocation

Translocation of NFAT in CFP-NFAT1 and R-GECO, WT–R-GECO or G652A–R-GECO co-transfected HEK293 cells was observed using an inverted microscope (Olympus IX71, Vienna, Austria) equipped with a 40 × 1.3 NA oil immersion objective. During the recordings using Live Acquisition v2.6 software (TILL Photonics FEI Company, Graefelfing Germany), the excitation of CFP was achieved using 430/20 nm filter and fluorescent images were captured using 483/32 nm emission filter (Semrock, Rochester, USA) with an ORCA-03G digital CCD camera (Hamamatsu, Herrsching am Ammersee, Germany). ImageJ 1.51n (NIH, Bethesda, USA) software was used to measure the fluorescence intensity in the nucleus and cytoplasm before and 50 min after stimulation with 10 µM OptoBI-1. These values (nucleus/cytosol) were then plotted using the SigmaPlot 14.1 software (Systat Software Inc., Erkrath, Germany).

### 2.6. Statistical Analysis

Data analyses and graphical display were performed using Clampfit 11 (Axon Instruments, Munich, Germany) and SigmaPlot 14.1 (Systat Software Inc., Erkrath, Germany). Data are presented as mean values ± S.E.M. Primarily, a Shapiro–Wilk test was conducted to test for the normality of the value distribution. Whenever normal distribution criterion was met, we used Student’s two-sample t-test or paired t-test to analyze the statistical significance. In general, differences were considered significant at *p* < 0.05 and indicated for individual comparisons in figures (* *p* < 0.05, ** *p* < 0.01, *** *p* < 0.001).

## 3. Results

### 3.1. TRPC3 Activity Pattern Generated in HEK293 Cells by Photopharmacology and Optochemical Genetics

In the first step we explored suitability of the G652A gain of function mutation for tuning of photocycling-induced TRPC3 activity pattern in HEK 293 cells. To do so, we set out to compare the temporal pattern of cation conductance generated by OptoBI-1 (10 µM)-mediated activation/deactivation cycling for TRPC3 wild-type and mutant channels. As shown in Figure 1A,B, HEK293 cells expressing WT-YFP or G652A-YFP channels were detected by their fluorescence and displayed about similar initial levels of basal current in the presence of *trans* OptoBI-1 (in the dark). Upon *cis* photoisomerization (illumination at 365 nm; UV light), cation conductances recorded with voltage-clamp increased rapidly and declined to basal (mutant) or even below basal levels (WT-YFP) channels upon *trans* isomerization (illumination at 430 nm; blue light; Figure 1B 430 nm/OptoBI). A significantly larger peak current amplitude (about 2–3 fold) was recorded during *cis* isomerization (illumination at 365 nm; UV light; Figure 1B 365 nm/OptoBI) in cells expressing the mutant channel. Interestingly, the latter difference vanished during repetitive (three times) photocycling, as evident from Figure 1A. Hence, mutant channels featured substantially larger initial peak current amplitudes associated with profound desensitization during repetitive Opto-BI-1 photocycling. Of note, the Ca^2+^/monovalent permeability ratios, as derived from reversal potentials (Figure 1A insert) in bionic conditions with Cs^+^ and Ca^2+^ as only cations in intracellular and extracellular solutions, were not significantly different (3.06 and 2.9 for WT and G652A mutant channels, respectively). Nonetheless, we detected another significant difference between WT and G652A currents. As shown in the left panel of Figure 1B DARK/No OptoBI-1, constitutive activity in the absence of the photochromic benzimidazole activators was barely detectable for the mutant channel, and basal activity of the mutant was apparently restored in the presence of OptoBI-1 in the dark. This may be interpreted in terms of the higher benzimidazole sensitivity of the mutant channel lacking constitutive activity. The moderate stimulatory effect of OptoBI-1 in the dark may be explained by either the presence of a small fraction of *cis*-OptoBI-1 in dark, or alternatively by partial activation of channels by *trans* OptoBI-1, which has been suggested previously [16]. The latter concept is supported by the observation that the benzimidazole-induced increase in basal activity of the G652A-YFP mutant was not abolished even during photoconversion of *cis*-OptoBI-1 back to *trans* via with blue light illumination (430 nm; Figure 1B 430 nm/OptoBI). By contrast, WT channels responded to blue light illumination with a decline in constitutive activity to a level significantly lower than the one initially observed in the absence of the photochromic benzimidazole (Figure 1B DARK/OptoBI). This finding suggested that WT-YFP may be controlled by OptoBI-1 in a dual manner with activation of the channel by *cis* photoisomerization and moderate, but significant inhibition to a level below its constitutive activity by blue-light-induced *trans* isomerization, as already suggested by Tiapko et al. 2019. Hence, we were able to produce temporally well-controlled TRPC responses with distinctly different basal and peak current amplitudes, which were expected to generate divergent patterns of local and global Ca^2+^ signals in HEK293 cells.

### 3.2. Light-Controlled TRPC3 Ca^2+^ Signaling Patterns and NFAT Nuclear Translocation in HEK293 Cells

**Figure 2:**
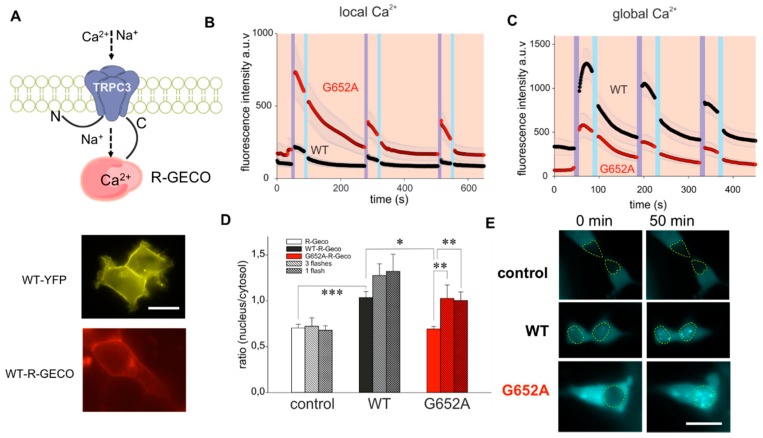
All-optical recording of TRPC3 Ca^2+^ signaling signatures suggests coupling between bulk cytosolic Ca^2+^ changes and NFAT1 activity. (**A**) Upper panel: Schematic presentation of TRPC3 C-terminal fusion with genetically encoded Ca^2+^ sensor (R-GECO). Lower panel: Fluorescence images of WT-YFP and WT-R-GECO fusions. Local (**B**) and global (**C**) OptoBI-1 (10 µM) photocycling-induced Ca^2+^ changes in HEK293 cells expressing WT-R-GECO and G652A-R-GECO (local) or WT-YFP and G652A-YFP with R-GECO (global), respectively. Time courses (mean ± SEM) of R-GECO fluorescent intensity during photoactivation of WT (white, WT-R-GECO: *n* = 15 cells; WT-YFP: *n* = 15 cells) and G652A (red trace, G652A-R-GECO: *n* = 20 cells; G652A-YFP: *n* = 15 cells). *Cis-trans* OptoBI-1 photoisomerization was repeated three times illuminating with UV (365 nm, 10 sec; violet) and blue light (430 nm, 10 sec; blue). (**D**) NFAT1 nuclear translocation in HEK293 cells overexpressing only CFP-NFAT1 and R-GECO (control; *n* = 11), WT–R-GECO (WT; black/grey, *n* = 11) or G652A–R-GECO (G652; red, *n* = 12). Mean ± SEM nucleus/cytosol fluorescence intensity ratio. CFP-NFAT1 translocation was monitored during OptoBI-1 photoconversion, with either three (3 Flashes) or one (1 Flash) photocycles of illumination as indicated. Statistical significance was tested by One-Way ANOVA (Holm-Sidak test for normally distributed values) or One-Way ANOVA for Ranks (Dunn’s test for non-normally distributed values), * *p* < 0.05, ** *p* < 0.01, *** *p* < 0.001, if not indicated – not significant (*p* > 0.05); (**E**) Images of NFAT1 translocation in HEK293 cells expressing CFP-NFAT1 only (control; upper panel) or co-expressing CFP-NFAT1 plus WT–R-GECO (WT; middle panel) or G652A–R-GECO (G652A; lower panel) before (0 min) and after (50 min) three cycles of UV (365 nm, 10 sec) and blue light (430 nm, 10 sec) illuminations. Nuclear regions are indicated. Scale bar represents 5 µm.

In the second step, we investigated the impact of amplitude modulation of TRPC3 activity on the generation of local and global Ca^2+^ signals in HEK293 cells. With the photocycling protocol shown in Figure 1A, we elicited three temporally identical rises in cation conductance and Ca^2+^ entry. Each activating pulse (10 s of 365 nm and 30 s 577 nm) lasted 40 s followed by a 40 s interval of channel deactivation induced by blue-light illumination (10 s of 430 nm and 30 s 577 nm). When WT-YFP channels were expressed, the series of activating light pulses generated transient rises of about equal peak amplitudes in cation conductance and returned to a conductance level significantly lower (*p* < 0.05) than constitutive activity during blue light-induced channel deactivation (Figure 1B 430 nm/OptoBI). With G652A-YFP mutant channels expressed, the first activating pulse displayed a substantially larger amplitude (*p* < 0.01) compared to WT followed by declining current responses to next to UV light applications, comparable to that obtained with WT channels. For a comparative evaluation of the linkage between these activity patterns and local Ca^2+^ levels within the vicinity of the TRPC3 channel, as well as to changes in global cellular Ca^2+^, we recorded Ca^2+^-sensitive fluorescence from R-GECO fused to the C-terminus of the channel proteins as a local reporter (WT-R-GECO; Figure 2A), and in parallel from soluble cytosolic R-GECO reporting global signals. As shown in Figure 2B, local Ca^2+^ signal recorded in this all-optical experimental setting, resembled the pattern of cation conductances measured by electrophysiology (Figure 1A,B). Local Ca^2+^ during the initial activating pulse was substantially higher with G652A-R-GECO channel expressed (*p* < 0.01), and this response declined profoundly during subsequent photocycling (Figure 2B). In cells expressing WT-R-GECO channels, fluorescence signals from the channel-linked R-GECO were essentially small with peak increase amounting to only about 25% of that generated with the mutant channel. These local Ca^2+^ signals declined further during subsequent photocycling pulses. The recording of global Ca^2+^ signals revealed a strikingly different picture. Photocycling of WT-YFP co-expressed with R-GECO channels triggered large cytosolic Ca^2+^ changes initiated from a rather high level of constitutively-elevated Ca^2+^, and the amplitude of cytosolic Ca^2+^ signals declined only moderately during subsequent photoactivation (Figure 2C). Global, transient Ca^2+^ rises triggered by photocycling of the G652A-YFP channel were significantly smaller than with WT channels (*p* < 0.05). The G652A-YFP Ca^2+^ transients started out at a low constitutive level with peak responses declining more profoundly during subsequent activation/deactivation cycling.

Hence, by utilizing photopharmacological control over TRPC3 activity, we were able to produce two distinctly different Ca^2+^ signaling patterns in HEK293 cells. Expressing WT generated constitutively elevated cytosolic Ca^2+^ levels and large transient increases in global cellular Ca^2+^ during photoactivation. By contrast, in cells expressing the PLC-insensitive G652A mutant of TRPC3, basal cellular Ca^2+^ levels remained low and photocycling of the mutant TRPC3 channel resulted in large local Ca^2+^ signals at the channel itself, while global Ca^2+^ was only moderately affected (Figure 2B,C).

**Scheme 1:**
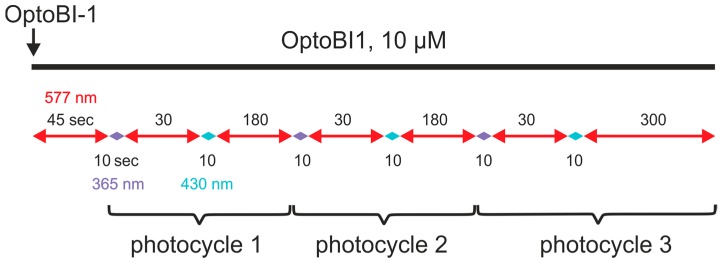
Schematic representation of OptoBI-1 application and illumination during [Ca^2+^]_i_ imaging. OptoBI-1 was added before and kept during the whole recording. Light illumination is cycled as indicated in the Scheme 1: Red arrow (577 nm)—detection of R-GECO fluorescence, violet (365 nm)—photoconversion of *trans*-OptoBI-1 in *cis*-OptoBI-1, blue (430 nm)—photoconversion of *trans*-OptoBI-1 in *cis*-OptoBI-1. Timing of each photocycle as indicated.

These two distinct Ca^2+^ signaling signatures were tested for an impact on NFAT1 nuclear translocation by expressing CFP-NFAT1 along with WT-R-GECO or the G652A-R-GECO mutant, respectively, and adopting the above-described protocol of repetitive photoactivation (three flashes of UV illumination) delineated in detail in Scheme 1. The extent of nuclear translocation of NFAT1 was quantified 50 min after initiation of the photoactivating pulses and a set of experiments was carried out, in which only a single activation/deactivation cycle was administrated (one flash of UV illumination). Ca^2+^-mediated activation of NFAT1 was quantified as the nuclear to cytosolic fluorescence ratio. As illustrated in Figure 2D, control levels remained at about 0.7 in all conditions in sham-transfected cells lacking WT/mutant overexpression. In cells expressing WT-R-GECO, a constitutively enhanced (about 1.0) level of cytosolic to nuclear distribution of the NFAT1 construct was detected (*p* < 0.001) and photocycling further increased this distribution to a ratio of about 1.3 (*p* < 0.05). This effect is illustrated by a video, documenting the time-dependent redistribution of NFAT1 fluorescence during the experiment (see Video S1 supplementary information). By contrast, cells expressing the benzimidazole supersensitive and basal activity-devoid mutant channel, displayed low resting NFAT activity with a relative nuclear/cytosolic distribution of about 0.7, which was elevated significantly to about 1.0 (*p* < 0.01) during photoactivation of the G652A-R-GECO. These effects on NFAT1 nuclear translocation were independent of the number of subsequent activating light pulses administration (Figure 2D three flashes/one flash). Overall, the effects of optical activation/deactivation of TRPC3 channels on NFAT1 localization correlated best with the observed global Ca^2+^ signals (Figure 2C). Moreover, our results indicated a particular signaling role of constitutive activity of WT, presumably due to the modest increase in bulk cytosolic Ca^2+^ levels.

### 3.3. Photopharmacological Suppression of Constitutive NFAT Nuclear Translocation in HEK293 Cells

Since both our electrophysiological and Ca^2+^ imaging results suggested that OptoBI-1 might exert an inhibitory action on the constitutive activity of TRPC3 upon repetitive *trans* photoisomerization (illumination at 430 nm), we hypothesized that OptoBI-1 might be suitable for light-mediated suppression of constitutive TRPC3-mediated NFAT activation.

**Figure 3:**
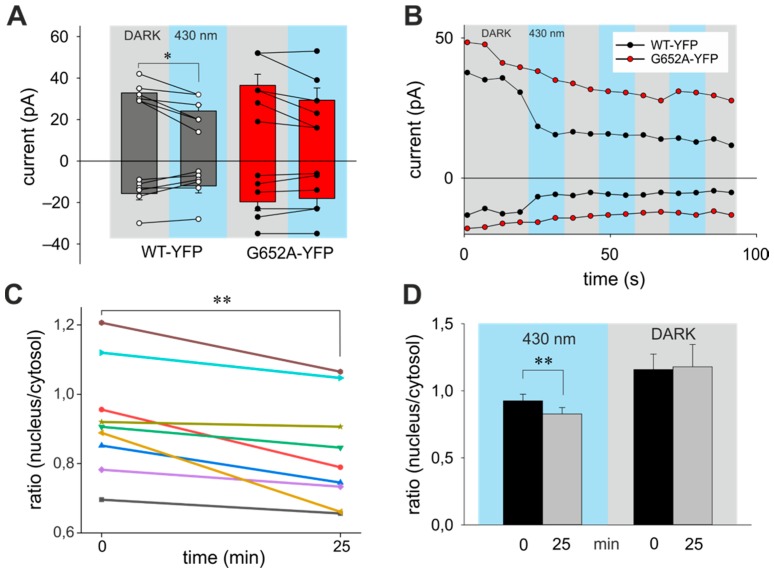
*Trans* photoisomerization of OptoBI-1 inhibits TRPC3 channel constitutive activity and basal NFAT1 nuclear localization. (**A**) Constitutive current levels of WT-YFP and G652A-YFP in presence of OptoBI-1 (10 μM) during blue light administration. Currents are shown for −90 and 70 mV (mean ± SEM). Statistical significance was tested by two tailed t-test (normally distributed values) or Mann–Whitney tests (non-normally distributed values), if not indicated—not significant (*p* > 0.05); (**B**) Representative time courses of the WT-YFP (white) and G652A-YFP (red) conductances recorded at −90 and 70 mV during repetitive photoconversion of OptoBI-1 (10 μM). Light illumination cycling is indicated as grey (DARK) and blue (430 nm); (**C**) nucleus/cytosol NFAT1 distribution responses are shown for individual cells. Fluorescent levels before (0 min) and after (25 min) blue light-induced changes in cells transfected with WT-R-GECO in presence of OptoBI-1 (10 μM; mean ± SEM); (**D**) mean ± SEM of nucleus/cytosol ratio NFAT1 translocation in WT-R-GECO-expressing HEK293 cells induced by OptoBi-1 non-illuminated (DARK) and illuminated with blue light stimulation (430 nm).

Consequently, we tested whether the constitutive level of NFAT1 nuclear translocation, as observed in TRPC3 overexpressing cells, is sensitive to inhibition by benzimidazole photopharmacology. To do so, we determined the consequences of exposing WT-YFP and G652A-YFP expressing HEK293 cells to both OptoBI-1 and blue light illumination. As shown in Figure 3A,B, blue light illumination indeed exerted an acute, rapid inhibition of basal TRPC3 conductance (*p* < 0.05). This effect was not detectable with the G652A mutant and confirmed the observation made in the activating/deactivating photocycling experiments shown in Figure 1B. Next, we tested suppression of WT basal activity with *trans*-OptoBI-1 in terms of NFAT1 translocation. When NFAT1 nuclear/cytosolic localization ratios were quantified after a 25 min period of repetitive blue light administration (10 s of blue light exposure every 30 s), the ratios dropped significantly (from 0.96 to 0.89; *p* < 0.01). This effect was not observed in WT-R-GECO-expressing cells that were exposed to OptoBI-1 without light stimulation (Figure 3D).

Our results demonstrate, for the first time, dual control over NFAT1 signaling based on photopharmacological targeting of TRPC3 channels.

## 4. Discussion

Linkage of TRPC channels to NFAT-mediated transcriptional control has been proposed repeatedly as a key mechanism of maladaptive tissue remodeling in the cardiovascular system [20,21]. The mechanistic principles by which TRPC signaling is decoded into NFAT nuclear translocation are incompletely understood, and NFAT activation by recombinant TRPC3 channels so far have not been demonstrated consistently. Here we provide evidence for coupling between the activity of recombinant TRPC3 channels and NFAT1 localization by utilizing precise control of channel activity by TRPC photopharmacology and chemogenetics. Direct, light-mediated control over activation and deactivation of TRPC3 wild-type and mutant channels (G652A), which displays supersensitivity to benzimidazoles and reduced basal activity [17,18], revealed a close relation between constitutive TRPC3 activity and NFAT1 signaling. This concept was supported by the observation of constitutive NFAT1 activity exclusively in cells overexpressing WT but not in cells overexpressing G652A mutant. Moreover, repetitive *trans* isomerization of the photochromic TRPC modulator OptoBI-1 was found to suppress TRPC3 constitutive activity, and this intervention reduced basal NFAT1 nuclear accumulation in cells overexpressing TRPC3. The molecular mechanism of channel inhibition by which rapid *trans* isomerization of OptoBI-1 via blue light irradiation is not clear as yet. Nonetheless, our previous investigations suggested that OptoBI-1 is bound by TRPC with submicromolar affinity in an isomer independent manner, with channel function being most likely modulated by photoisomerization of OptoBI-1 molecules, which presumably remain coordinated within the channel complex [16]. Our current study demonstrates that UV light-induced *cis* photoisomerization of OptoBI-1 triggers profound activation of mutant channels, as expected from the described benzimidazole supersensitivity of the mutant. The large cation conductance generated by the mutant channel was consistently associated with large local Ca^2+^ signals, as evidenced by a Ca^2+^ reporter (R-GECO) fused to the channel’s cytosolic domain. Of note, the G652A mutation did not significantly affect the channel’s Ca^2+^ permeability as estimated from a determination of the Ca^2+^/Cs^+^ permeability ratios, which were found not significantly different between WT and mutant channels. Hence the larger local Ca^2+^ signals generated by the mutant were based mainly on its large open probability in the presence of benzimidazole agonists [17]. Interestingly, these large inward currents and associated Ca^2+^ entry mediated by the mutant channel were only modestly translated into global cytosolic Ca^2+^ signals and associated with small albeit distinct NFAT1 activation. By contrast, expression of recombinant WT channels in HEK293 cells, in spite of generating rather small cation currents and local Ca^2+^ signals in the vicinity of the channel, increased basal levels of global Ca^2+^ allowing the accumulation of NFAT1 within the nucleus. Photoactivation of WT channels triggered large cytosolic Ca^2+^ changes and further promoted NFAT1 nuclear translocation. The principle underlying the more effective coupling of WT TRPC activity to global cytosolic Ca^2+^ homeostasis as compared to the mutant channel displaying higher activator sensitivity are still unresolved. Although we did not directly measure the responses to light manipulation at the level of gene expression, due to difficulties to completely reconstitute the signaling pathway by heterologous overexpression, our results suggest the principal feasibility to manipulate NFAT transcriptional activity by TRPC photopharmacology. Moreover, we demonstrate that recombinant TRPC channels couple to NFAT1 activity via global rather than local microdomain Ca^2+^ signaling. In this aspect, recombinant TRPC channels clearly differ in their mechanistic coupling to NFAT signaling as compared to the STIM/Orai pathway, which does not require bulk Ca^2+^ changes for downstream coupling to calcineurin/NFAT signaling [22]. Our observation that only a single photocycling trigger was sufficient to initiate or promote NFAT1 activation, while modification of the temporal activation pattern (repetitive activation) lacked any further impact on the NFAT signal, is consistent with the reported temporal uncoupling of NFAT1 transcriptional activity from the upstream Ca^2+^ signal owing to the slow processes controlling its nuclear export and off rate [23].

Of note our results were obtained with recombinant TRPC3 channels and NFAT1 expressed in HEK293 cells. We are aware that conclusions drawn from these reconstitution experiments are of certainly limited transferability with respect to native tissues. Nonetheless, principle mechanistic linkage of signaling molecules as well as basic concepts of pharmacological intervention can be conclusively demonstrated. Specifically, the here reported coupling between basal TRPC3 activity and constitutive NFAT1 activation may represent a mechanism involved in pathological transitions in human tissues, which are associated with elevated TRPC3 levels and distorted Ca^2+^ transcription coupling such as cardiovascular remodeling [20,21].

## 5. Conclusions

In aggregate, our study demonstrates the principle suitability of TRPC photopharmacology to manipulate NFAT transcriptional activity with high spatiotemporal precision. Our experiments with recombinant TRPC3 channels suggest efficient coupling of TRPC constitutive activity to global Ca^2+^ homeostasis and NFAT1 transcriptional activity. This mechanism may be of relevance in tissues featuring enhanced TRPC3 expression associated with pathological remodeling processes and is demonstrated to be sensitive to TRPC3 channel inhibition by benzimidazole photopharmacology.

## Supplementary Materials

The following is available online at https://www.mdpi.com/2073-4409/9/3/556/s1, Video S1: Video of NFAT1 translocation in HEK293 cells co-expressing CFP-NFAT1 plus WT–R-GECO before and after three cycles of UV (365 nm, 10 s) and blue light (430 nm, 10 s) illuminations.
